# Eyes on me: Investigating the role and influence of eye-tracking data on user modeling in virtual reality

**DOI:** 10.1371/journal.pone.0278970

**Published:** 2022-12-29

**Authors:** Dayoung Jeong, Mingon Jeong, Ungyeon Yang, Kyungsik Han

**Affiliations:** 1 Department of Artificial Intelligence, Hanyang University, Seoul, Republic of Korea; 2 Electronics and Telecommunications Research Institute, Daejeon, Republic of Korea; Karunya Institute of Technology and Sciences, INDIA

## Abstract

Research has shown that sensor data generated by a user during a VR experience is closely related to the user’s behavior or state, meaning that the VR user can be quantitatively understood and modeled. Eye-tracking as a sensor signal has been studied in prior research, but its usefulness in a VR context has been less examined, and most extant studies have dealt with eye-tracking within a single environment. Our goal is to expand the understanding of the relationship between eye-tracking data and user modeling in VR. In this paper, we examined the role and influence of eye-tracking data in predicting a level of cybersickness and types of locomotion. We developed and applied the same structure of a deep learning model to the multi-sensory data collected from two different studies (cybersickness and locomotion) with a total of 50 participants. The experiment results highlight not only a high applicability of our model to sensor data in a VR context, but also a significant relevance of eye-tracking data as a potential supplement to improving the model’s performance and the importance of eye-tracking data in learning processes overall. We conclude by discussing the relevance of these results to potential future studies on this topic.

## 1 Introduction

One method that has attracted significant attention in recent VR research involves using the data collected from the sensor equipment worn by the user as a way to analyze various aspects of their state or behavior during the VR experience. For example, studies have examined changes in the user’s eye movement (e.g., focus point, gaze movement, pupil diameter) based on the data collected from an eye-tracking device, those in their head or body movements collected from head-mounted displays (HMD) or trackers, and those in their electrodermal activity (EDA) collected from wearable devices [1–6]. Studies have also attempted to understand the relationships between the collected sensor data and user-driven factors, such as stress, engagement, satisfaction, and conversation quality, through statistical analysis and model development [7–11]. Findings from these studies not only help determine whether the goal of a given VR system has been achieved but also provide design insights that can be used to improve the user experience and the system itself.

When collecting data during a VR experience, it is especially important to measure and monitor human states or behaviors as unobtrusively as possible. Hence, collecting signals generated from standard devices (e.g., HMD) or from devices that do not cause the user discomfort is essential. It is worth noting that many studies have been investigating eye-tracking primarily because visuals are the first channel of information collection from a human [12, 13]. The use of eye-tracking data in research is also increasing because eye-tracking devices have become more affordable in recent years. Eye-tracking is key to human processing of visual information as well as measuring attention, interest, and arousal. In other words, eye-tracking data can be used to digitize the ways in which people communicate with computers, allowing researchers to identify and analyze patterns of visual attention of individuals as they perform specific tasks (e.g., reading, searching, driving, scanning an image). Perhaps due to such important roles, eye-tracking data has been used for different purposes, including intention recognition [14], image classification [15–18], depression recognition [19], and attention prediction [20].

Another group of research has focused on developing prediction models. Studies have employed machine learning algorithms (e.g., Support Vector Machine (SVM), Logistic Regression, Random Forest, *k*-Nearest Neighbors, Na ve Bayes), and more recent studies have begun to utilize deep learning methods (Convolutional Neural Network (CNN) and Long Short-Term Memory (LSTM)) to extract deep features of the data and construct a more complicated learning models [18, 21, 22]. These studies have utilized various types of features extracted from sensor signals as the data for their prediction models and demonstrated high possibilities of predicting users’ states or behaviors through their models.

In this work, we aimed to achieve the same goal as prior studies, which is to measure and model a user’s state and behavior based on sensor signals, particularly in terms of eye-tracking data in the context of VR. Despite its demonstrated role in depicting human characteristics in prior studies, the adaption and study of eye-tracking data in VR has been under-studied. Another limitation is a lack of consideration of measuring and analyzing a prediction model from multiple case studies, which possibly fails to suggest more generalizable results or insights. To fill these research gaps, this paper presents two independent case studies of cybersickness [23, 24] and locomotion [25], both of which are closely related to the VR user experience. Our two case studies leverage various sensor signals generated from an HMD, a tracker, and a physiological device (e.g., head, eye, and ankle movement, electrodermal activity (EDA)) for the development of two deep learning models that classify the level of cybersickness and the type of locomotion, respectively. The model was designed to learn not only characteristics of the individual sensor data modalities through an *attention* technique (i.e., calculating different degrees of the importance of data features during training) [26] but also the *temporal sequence* of the sensor data. The same model architecture was applied to both studies as our intention was to examine a possible generalization of the role of eye-tracking data in understanding a user’s state and behavior. For the same purpose, we recruited enough participants for each study comparable to prior studies [27, 28]. The cybersickness study involved 27 participants, and the locomotion study was conducted with 23.

The experiment results of two studies were quite similar. Both studies showed that the addition of eye-tracking data increased the performance of the model, which was at its highest when all sensor modalities were used. These results demonstrated the effectiveness of both our model structure and the use of eye-tracking data as a supplemental method to improve the prediction performance.

In summary, this paper makes the following contributions.

- We proposed a deep learning-based architecture for modeling a user’s state and behavior (cybersickness and locomotion), based on sensor data in VR.
- We identified a role for eye-tracking data signals in modeling users in VR.

## 2 Related work

### 2.1 Research using eye-tracking data

Eye-tracking data is generated based on the movement of the pupils. Some devices that can take these measurements include Tobii (https://www.tobii.com/) and Pupil labs (https://pupil-labs.com/). Recent HMD devices, such as HTC VIVE Pro Eye (https://www.vive.com/us/product/vive-pro-eye/overview/) and FOVE (https://fove-inc.com/), provide eye-tracking components at affordable prices, making the utilization of eye-tracking data in a VR environment more practical and relevant for researchers and practitioners.

Research utilizing eye-tracking data has been actively conducted in non-VR contexts. One of the topics that has been widely studied is image classification. To name a few, Ahmed and Noble [15] used eye fixations to classify and acquire the image frames of head, Zhou et al. [29] used fixations and region of interest (ROI), Karessli et al. [16] used gaze points, Saab et al. [18] used gaze data, Roy et al. [17] used eye fixations, fixation duration, pupil diameter, and polar moments to develop a cognitive model for ambiguous image classification. Other classification topics include intention recognition [14], attention prediction [20], depression recognition [19], and reading pattern detection [30]; similar types of eye-tracking features were used.

Eye-tracking in a VR context also provides researchers with opportunities [31] to accurately control the VR environment through ROI settings, which helps enhance the user experience by providing a more thorough understanding of the interactions between the user and the objects inside the VR environment [1–3, 32, 33]. For example, Lahiri et al. [3] developed a VR system that supports social skill improvement for people with autism. Pfeiffer et al. [33] studied the shopping motives of users in a VR environment. While eye-tracking features are identical regardless of whether they were collected and studied in non-VR or VR conditions, differences in the environment from which they were collected are quite different. VR is a virtual world that can be manipulated depending on its goals or tasks; as such, the characteristics and interpretations of sensor signals associated with users’ experiences and interactions in such an environment may be different from those in a non-VR environment. In this sense, we aim to examine the role of eye-tracking data in VR contexts and discuss ways to utilize it to enhance user experience.

### 2.2 Research on prediction model with eye-tracking data

Most studies described in the previous section defined various classification models and their performances, highlighting the role and influence of eye-tracking data [34, 35] on characterizing users and tasks in specific scenarios. Some of the most widely used eye-tracking features include pupil diameter, pupil position, eye fixations, blinking, and gaze point. Various types of classification algorithms, including Support Vector Machine (SVM), *k*-Nearest Neighbors (kNN), Decision Trees, Logistic Regression, and Na ve Bayes have been employed to track this data. SVM appears to be the most promising [34, 35]. Recent studies have begun to employ deep learning algorithms [18, 36], such as CNN and LSTM, but the number of deep learning-based modeling research is smaller than that of machine learning-based one. As deep learning models have shown greater performances than machine learning models in many domains, we expect to see more studies on learning user characteristics through deep learning algorithms in VR domains. Given that a large volume of sensor data is generated in VR, deep learning algorithms have the potential to play a key role in modeling and understanding users.

The goal of our research is also to utilize eye-tracking data to model a user’s state or behavior as we found that the utilization of eye-tracking data in a VR context is quite under-studied. In this paper, we present a study that offers additional insights into the way in which eye-tracking data can be collected and used. We present a deep-learning model using *attention*, one of the learning mechanisms employed to calculate different degrees of the importance of data features during training. Our model also considers *temporal sequences* of the data in order to more accurately capture a user’s state and behavior. Furthermore, we present findings from the application of the model to two specific case studies (one is cybersickness and the other is locomotion). The demonstration of the use of our model in two different case studies also highlights the robustness of our proposed multimodal, attention-based deep learning model in terms of its potential use and application in other VR/AR scenarios as well.

## 3 Case study backgrounds

**Fig 1:**
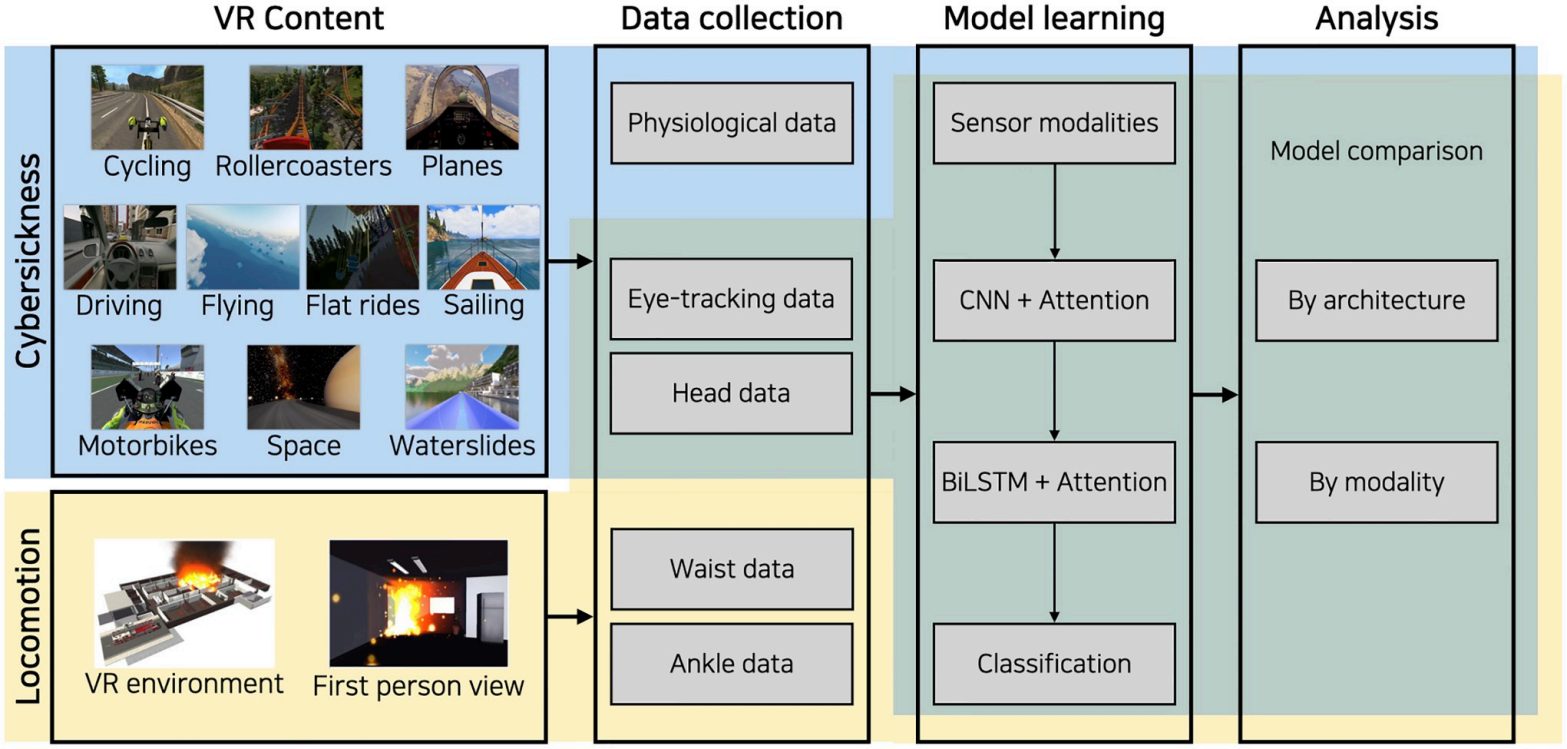
The study procedure. Each study has its own VR content, providing participants with the proper environment for cybersickness or locomotion. We collected eye-tracking, head, and physiological data from the cybersickness study and collected eye-tracking, head, waist, and ankle data from the locomotion study. We used the same model architecture and analysis methods for both studies.

Fig 1 illustrates the overall procedure, which was the same for both studies. Two studies collected different types of data to define their participants’ experiences. Eye-tracking, head, and physiological data were collected from the cybersickness study while eye-tracking, head, waist, and ankle data were collected from the locomotion study. The model learning and analysis phases were the same for both studies. Our study was reviewed and approved by the internal institutional review board at the authors’ university (Hanyang University, HYUIRB-202209-003). We recruited participants over the age of 19 and obtained written informed consent from all participants in the experiment.

### 3.1 VR sickness (cybersickness)

#### 3.1.1 Study background

Cybersickness is one of the main degrading factors that of the VR/AR experience. One possible way to alleviate this involves developing a technology that can predict and respond to cybersickness based on data collected from the environment. Various body-related data (e.g., eye tracking, head movement, body movement) from an HMD or a tracker as well as biometric data (e.g., skin conductance, heart rate) from additional wearable sensing devices can be used to determine each factor’s association with cybersickness. As more effective and advanced artificial intelligence algorithms that learn the characteristics of time-series sensor data continue to develop, it is expected that sensor signals can be utilized in cybersickness prediction.

#### 3.1.2 Study procedure

As individuals may feel different levels of cybersickness, we prepared videos in various topics. Thus, we prepared 20 VR videos with 10 categories (each category comprised two videos), namely cycling, driving, flat rides, flying, motorbikes, planes, roller coasters, sailing, space travel, and water slides (as shown in Fig 1).

We used the HTC VIVE Pro Eye and the Empatica E4 wristband (https://www.empatica.com/research/e4/) for the experiment and played the VR videos in VR environments (made with Unity 3D) in a Windows 10 PC with Intel Core i7 and GeForce RTX 2070.

We recruited 27 participants via university bulletin boards and word-of-mouth. The experiment consisted of two phases: (1) answering a survey and (2) watching VR videos. Before the experiment began, we explained the goal and procedure of our study to each participant and let them know that they could opt-out anytime. We then obtained informed consent from the participants.

First, each participant was informed of the goal and procedure of the study and asked to answer demographic questions (age, gender, and VR experience) and to complete the Motion Sickness Susceptibility Questionnaire (MSSQ) [37] before starting the experiment. The mean age was 26.2 (SD = 3.3) and 16 participants were male and 11 were female. 19 of the participants had previous VR experience, and the VR experience question offers yes or no answer options, following [38, 39]. MSSQ is designed to measure the user’s usual motion sickness level. It contains nine questions (with a five-point Likert scale) about whether a subject feels motion sickness from various riding conditions (e.g., cars, trains, swings). The higher the sum of the responses, the more likely the subject is to feel motion sickness. The average MSSQ score of our study participants was 10.0 (SD = 8.8), which is similar to those in [37] (mean: 12.9, SD = 9.9 from 148 participants) and [40] (mean: 9.8, SD = 7.9 from 12 participants). This means that our study participants were not different from those in other studies regarding MSSQ. Lastly, the participants were instructed to describe their condition upon experiencing severe cybersickness and were again assured that they could end their participation at any time. The participants were also provided with enough time to become familiar with the HMD and the E4 wristband.

**Fig 2:**
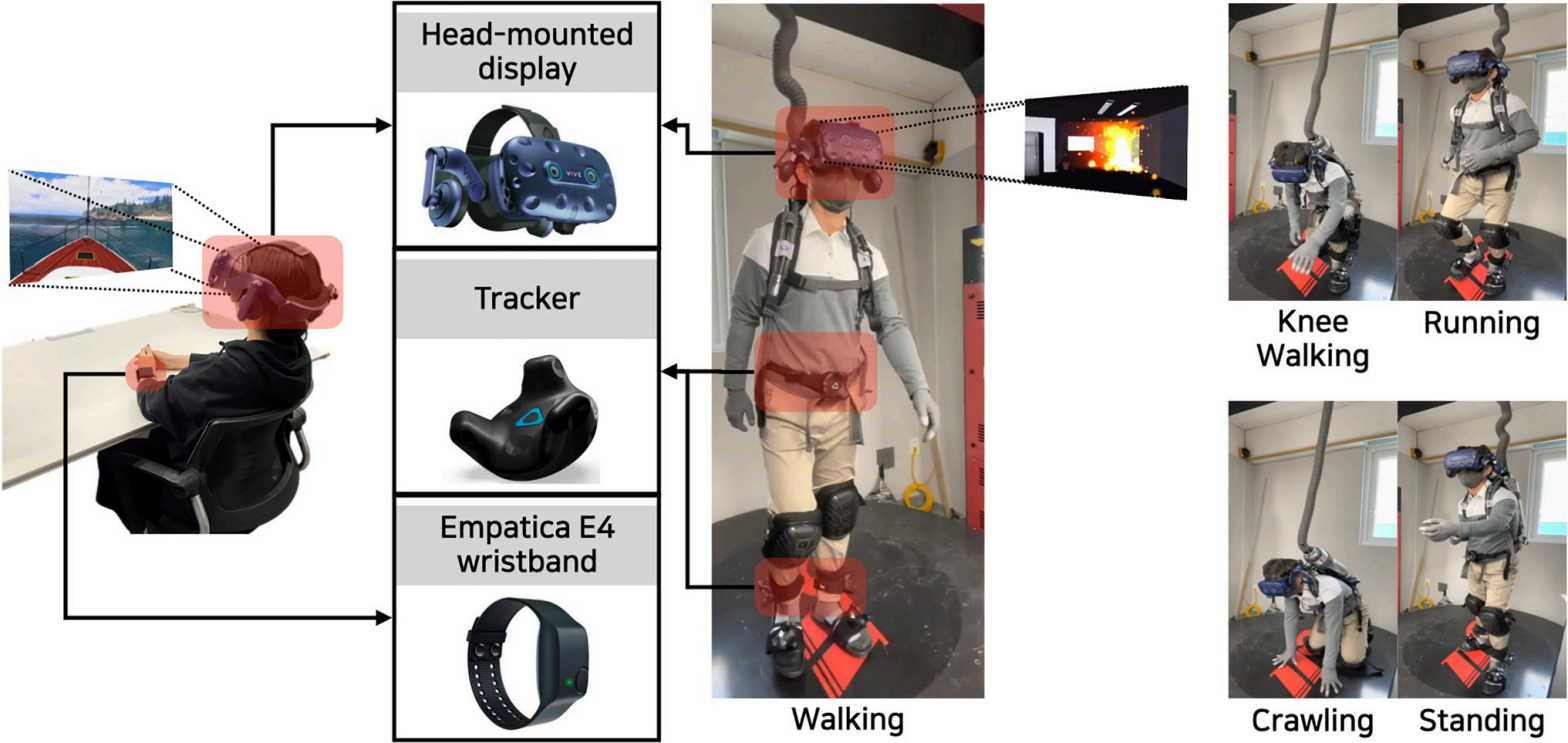
Data collection. Left: cybersickness study, Right: locomotion study.

Second, each participant was asked to sit on a chair and watch a VR video in a position that was comfortable for them (Fig 2-left). The video-watching phase consisted of four sessions. Five VR videos were played during each session, with each video running for 45 seconds, and the order of five videos was randomly chosen from different categories. Given that previous cybersickness studies used the videos that ran for around 30 seconds [41, 42], we believe that the length of the videos in our study was sufficient. The participants were given a 15-second break at the end of each video and a 7-minute break at the end of each session to minimize the effect of their experience in the previous video before watching the next one, following [27, 41]. They were also offered additional time to rest between videos and sessions. It took around 46 minutes per participant to complete the study. The participants who completed the experiment received a $10 gift card for their time and participation.

It is important to note that the participants may have had different degrees of inherent motion sickness. The representative scales for measuring cybersickness level are the Simulator Sickness Questionnaire (SSQ) [43] and the Fast Motion Sickness scale (FMS) [44]. The SSQ responds to 16 questions related to cybersickness symptoms, and each symptom is evaluated by dividing it into three factors (oculomotor, disorientation, and nausea). On the other hand, the FMS has a simpler evaluation method than SSQ. The user needs to choose a number between 0 (no sickness at all) and 20 (frank sickness). Prior studies have proven that the responses from the SSQ and from the FMS are highly correlated; thus many studies have used either question method depending on the design and goal of their experiment [27, 41, 45–51]. In this work, we employed the FMS which has the advantage of taking quick responses about each video. The participants were asked to indicate their level of cybersickness after watching each VR video via the FMS. In accordance with prior research [27, 47], we defined the level of cybersickness based on the quartiles which were determined based on the data from all participants. In our data, the first quartile (*Q*_1_) was 1.0, the second quartile (*Q*_2_) was 4.0, and the third quartile (*Q*_3_) was 7.0. The data was labeled as follows.

$$\begin{array}{c} Cybersickness\;level\;=\;\{\begin{array}{cc} \text{None} & \text{if}\;0\leq \text{FMS}\leq Q_{1}\\ \text{Low} & \text{if}\;Q_{1}<\text{FMS}\leq Q_{2}\\ \text{Moderate} & \text{if}\;Q_{2}<\text{FMS}\leq Q_{3}\\ \text{High} & \text{if}\;Q_{3}<\text{FMS}\leq 20 \end{array} \end{array}$$ (1)

Eye-tracking and head movements were collected using the HMD and physiological data was collected using the E4 wristband, both were set to a frequency of 30Hz. The eye-tracking data consisted of 23 features, including gaze direction (*x*,*y*,*z*) with both eyes integrated as well as gaze direction (*x*,*y*,*z*), gaze origin (*x*,*y*,*z*), pupil diameter, pupil position (*x*,*y*) and eye openness for each eye. The head movement data was made up of six features, including position and rotation data (*x*,*y*,*z*). We considered a single EDA feature as a physiological signal that is frequently done in VR research [27, 45, 52] (See Table 1). Overall, we collected a total of 24,300 seconds of data: 27 (number of participants) × 20 (number of videos) × 45 (one video time).

**Table 1:** Data features used in each study. Two studies used the same features from the HMD. The physiological sensor was used in the cybersickness study, and the features from the tracker were used in the locomotion study.

Data device	Cybersickness	Locomotion
HMD	head movement and orientation (*x*,*y*,*z*) gaze direction (*x*,*y*,*z*) with both eyes integrated gaze direction and origin (*x*,*y*,*z*) pupil diameter, pupil position (*x*,*y*) eye openness presented from right and left eyes	
Tracker	-	waist movement and orientation ankle movement and orientation all presented by *x*,*y*,*z* values
E4 wristband	electrodermal activity	-

### 3.2 VR locomotion

#### 3.2.1 Study background

Given that locomotion is body reactions, VR environments need to be able to support it if the corresponding VR scenarios involve user movement (e.g., walking, running, moving around). Locomotion research can be broadly classified into walking-, steering-, selection-, and manipulation-based techniques [53]; of these, the walking-based technique has been studied the most. In this technique, Walking-in-Place (WiP), which refers to step-like movement while remaining stationary [25], is best known. The user’s limb movements can be tracked, or stepping and treadmill-like input devices can be used, similar to our study environment (Fig 2-right), to register the step-like movements and translate them into VR motion.

#### 3.2.2 Study procedure

VR technology has been widely adopted to support virtual training in many scenarios [54–56]; as such, the context of our study of VR locomotion was firefighting training. This training involves many different types of movements that are expected to be supported by VR training; the locomotion types (walking, running, walking on one’s knees, crawling, standing) considered in our study were the ones that firefighters use during their missions. Walking and running were defined based on movement speed and magnitude. Kneeling forward and crawling on one knee are actions that lower one’s posture and move the body forward. Standing refers to a posture with the knee of the front leg slightly bent and the hind leg extended to support the weight. Our research team collaborated with local firefighters for the design and implementation of this VR training system, and one of their design requirements was the inclusion of such movements.

We recruited 23 participants (all firefighters) for the user study by sending official invitations to local municipal fire departments because the study was part of firefighting training system development. We believe that data collected by domain professionals (real firefighters) would increase the reproducibility of our data in other training scenarios (e.g., military training, safety training) in which locomotion data generated from professionals is important. The participants were invited to a living laboratory organized by a research institution and given instructions on how to use the VR devices and what to do in the VR environment. The mean and SD of the participants’ age were 24.3 and 2.6, respectively. All participants were male, and twelve of them had previous casual VR experience.

Fig 1 (the locomotion area colored in yellow) shows the virtual environment (a case of a fire disaster in a basement of a building) that we created for data collection, and Fig 2-right shows five types of locomotion that the participants performed. The participants were asked to wear an HMD, and VIVE trackers were attached to their waist and ankles. We asked the participants to follow the prompt (e.g., “walk forward for ten seconds”) on the HMD screen as naturally as possible when it appeared on the screen. After each prompt, the participants were given a five-second break before another message prompting the next locomotion type (e.g., “kneel and move forward for ten seconds”) popped up on the screen. One session includes five movement types and lasted about 70 seconds in total. We planned for three sessions during the experiment. Most of the participants (74%; 17 out of 23) completed three session, but some did up to five sessions as they wanted to allow for the collection of a sufficient amount of sensor data (305 seconds per participant on average, 7,020 seconds in total) that corresponded to each movement types. All sensor data was also collected at 30Hz, same as the cybersickness study. The participants who completed the experiment received a $10 gift card for their time and participation.

We used the *x*, *y*, and *z* coordinates of waist and ankle movement and orientation. Same as the cybersickness study, we used the same features of head movement, orientation, and eye-tracking data from the HMD.

## 4 Model development

**Fig 3:**
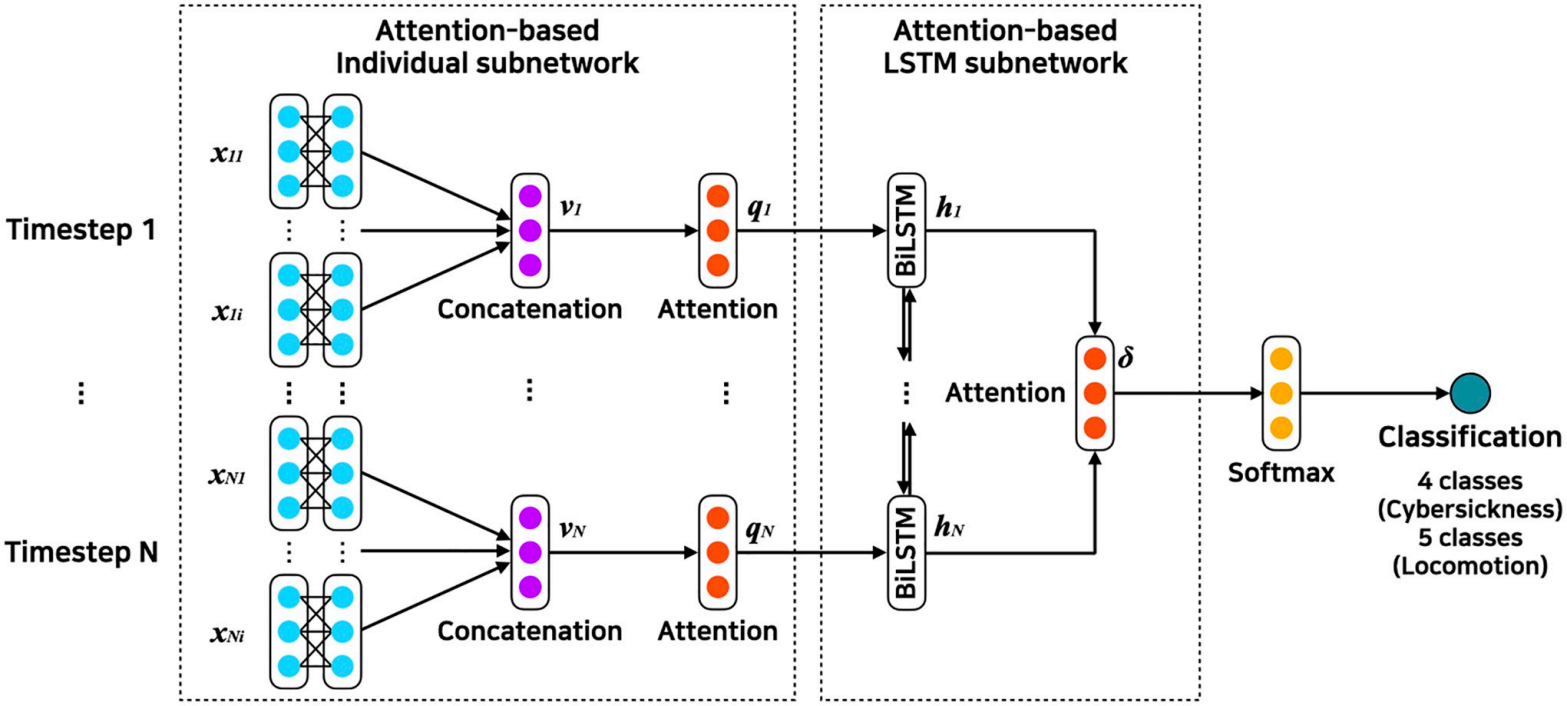
Model architecture used in two studies. The model mainly consists of the attention-based individual network and the attention-based LSTM subnetwork. The cybersickness study has four classes, and the locomotion study has five classes (*N* means time *t*, which is *t* = 1,2,…,*N*).

Our proposed model consists of three key components as follows: (1) an attention-based individual subnetwork that considers the relative importance of each data modality to fuse modality-specific features, (2) an attention-based LSTM subnetwork that extracts the importance of timestep and fuse the hidden state of the Bidirectional LSTM (BiLSTM), and (3) an output layer that uses a softmax function to obtain the probabilities for classification. Fig 3 illustrates the detailed architecture of our model.

### 4.1 Attention-based individual subnetwork

Each data modality has different feature sizes, thus, we first passed the raw features to Principal Component Analysis (PCA) [57] in order to have the same feature size for all data modalities. Individual convolutional subnetwork was used to extract features of each data modality. The frequency representation of the *i*^*th*^ sensor at time *t*, *x*_*ti*_, was passed to the convolutional subnetwork. Two convolutional layers with the activation function ReLU (Rectified Linear Unit) [58] were used in the subnetwork. Then a feature vector *v*_*ti*_ was generated and used as the input to the attention fusion subnetwork.

We employed an attention network to prioritize the importance of data modalities [59]. The network takes the feature vectors of data modality [*v*_*t*1_, *v*_*t*2_, …, *v*_*ti*_] as input and generates an attention weight for each modality. The hidden representation of *v*_*ti*_ was computed to get *μ*_*ti*_ with a sensor-level context vector *w*_1_.

$$\begin{array}{c} \mu_{ti}=tanh\left(W_{1}v_{ti}+b_{1}\right) \end{array}$$ (2)

Then a normalized weight *α*_*ti*_ was computed through a softmax function.

$$\begin{array}{c} \alpha_{ti}=\frac{exp({\left(\mu_{ti}\right)}^{T}w_{1})}{\sum_{i}exp\left({\left(\mu_{ti}\right)}^{T}w_{1}\right)} \end{array}$$ (3)

where *W*_1_, *b*_1_, *w*_1_ are parameters of the attention network. They are randomly initialized and jointly learned through the training phase. Then the vectors of all data modalities are fused by using their attention scores as weights in order to make a uniform feature representation vector *q*_*t*_.

$$\begin{array}{c} q_{t}=\sum_{i}\alpha_{ti}v_{ti} \end{array}$$ (4)

### 4.2 Attention-based LSTM subnetwork

The output [*q*_1_, *q*_2_, …, *q*_*N*_] is passed to a stacked LSTM structure [60]. LSTM is a recurrent neural network (RNN) architecture that remembers values over arbitrary intervals and deals with the vanishing gradient problem that can be encountered when training traditional RNNs.

$$\begin{array}{cc} & f_{t}=\sigma \left(W_{f}\cdot \left[h_{t-1},q_{t}\right]+b_{f}\right) \end{array}$$ (5)

$$\begin{array}{cc} & i_{t}=\sigma \left(W_{i}\cdot \left[h_{t-1},q_{t}\right]+b_{i}\right) \end{array}$$ (6)

$$\begin{array}{cc} & \tilde{C_{t}}=tanh\left(W_{C}\cdot \left[h_{t-1},q_{t}\right]+b_{C}\right) \end{array}$$ (7)

$$\begin{array}{cc} & C_{t}=f_{t}*C_{t-1}+i_{t}*\tilde{C_{t}} \end{array}$$ (8)

$$\begin{array}{cc} & o_{t}=\sigma \left(W_{o}\left[h_{t-1},q_{t}\right]+b_{o}\right) \end{array}$$ (9)

$$\begin{array}{cc} & h_{t}=o_{t}*tanh\left(C_{t}\right) \end{array}$$ (10)

where *f*_*t*_ is a forget gate, *i*_*t*_ is an input gate, and *o*_*t*_ is an output gate. *C*_*t*_ is a cell state. A hidden state *h*_*t*_ is generated at each timestep. Standard RNN (including LSTM) uses the last timestep as a single representation for the whole input sequence. This generally leads to less consideration of the front part of the sequence for classification. Because the hidden state at each timestep may show a different level of impact on classification (in our case, occurrence of cybersickness), we applied the attention mechanism again to calculate the weighted average sum of all hidden states.

Given all hidden states *H* = [*h*_1_, *h*_2_, …, *h*_*N*_] (*h*_*t*_ refers to a hidden state at timestep *t*), the attention for LSTM can be formalized as follows:

$$\begin{array}{cc} & \gamma_{t}=tanh\left(W_{2}h_{t}+b_{2}\right) \end{array}$$ (11)

$$\begin{array}{cc} & \beta_{t}=\frac{exp({\left(\gamma_{t}\right)}^{T}w_{2})}{\sum_{t}exp\left({\left(\gamma_{t}\right)}^{T}w_{2}\right)} \end{array}$$ (12)

$$\begin{array}{cc} & \delta =\sum_{t}\beta_{t}h_{t} \end{array}$$ (13)

where *w*_2_ is a time-level context vector, *β*_*t*_ is a normalized weight through a softmax function, and *δ* is the uniform representation of the whole sequence computed based on the sum of all hidden states. Each hidden state is updated by its attention weights. *W*_2_, *b*_2_, *w*_2_ are the parameters of the attention-based LSTM subnetwork which are randomly initialized and jointly learned during the training phase. We constructed a BiLSTM model that better learns the temporal characteristics of the data. BiLSTM has been found to be more efficient than unidirectional LSTM because it considers both past and future data through an interactive network [61].

### 4.3 Output layer

The output of attention-based LSTM subnetwork is calculated through an output layer using a fully-connected layer and a softmax function to predict cybersickness.

$$\begin{array}{c} prediction=\underset{a\in A}{argmax}\left(softmax\left(W_{3}\cdot \delta +b_{3}\right)\right) \end{array}$$ (14)

where *A* is the set of all data modalities. *δ* is transformed to the probability of each modality and the prediction result is determined by searching modality with maximum probability.

## 5 Experiment setup

For the experiment, we implemented our model in Pytorch and trained it on a server with GeForce RTX 2070.

First, for cybersickness, the data of each participant consists of a set *S* of different data modalities in the form of time series data *S*_*t*_ = {*E*_*t*_, *H*_*t*_, *P*_*t*_}, where *E*, *H*, and *P* refer to eye-tracking, head movement, and physiological data, respectively. For locomotion, *S*_*t*_ = {*E*_*t*_, *H*_*t*_, *I*_*t*_, *A*_*t*_} was used, where *I* and *A* refer to waist and ankle data, respectively.

Then, each item in *S*_*t*_ is divided into a set of *r* time windows $W_{t}^{a}=\left\{w_{t1}^{a},w_{t2}^{a},...,w_{tr}^{a}\right\}$ of a fixed length of *T*_*w*_ seconds (we set *r* = 30 and *T*_*w*_ = 1). *S*_*t*_ is then split into the training set, validation set, and test set with the ratio of 7:1:2 by chronological order.

Second, for model training, we used cross entropy for loss function and Adam for optimizer. The model was trained up to 500 epochs, and an early stop strategy was used with 20 times of patience. The best parameters of the model was selected through parameter tuning with the validation set (batch size = 64 and learning rate = 0.001). We used 5-fold cross validation for model training and used the macro F1-score as the metric for the performance evaluation of the model on the testing dataset. The macro F1-score is preferred over the micro F1-score, because it can evaluate the overall performance of the multi-classifier by giving equal weight to all classes [62, 63].

We measured the effectiveness of our model by comparing it with other models. The models that we considered for the comparison include SVM, CNN, BiLSTM, and CNN-BiLSTM that have been extensively used in cybersickness prediction in prior studies [27, 28, 41, 49, 64]. Our model has three variants including **A-INV** that has the attention network only in the individual subnetwork, **A-BiLSTM** that has the attention network only in the LSTM subnetwork, and our proposed model (**Ours**) that has the attention network both in the individual and LSTM subnetworks.

## 6 Results

### 6.1 VR sickness (cybersickness)

Table 2 summarizes performance results of the model for cybersickness. Our proposed model yielded the highest performance (0.82 F1-score) compared with other models. A-INV showed a better performance than A-BiLSTM (3% difference). Overall, the application of the attentions for the sensor modalities and the BiLSTM appeared to be effective.

**Table 2:** The model performance by architecture (cybersickness). Our model yielded the highest performance.

Model architecture	F1-score
SVM	0.57
CNN	0.52
BiLSTM	0.71
CNN-BiLSTM	0.68
A-INV (CNN-Attention-BiLSTM)	0.81
A-BiLSTM (CNN-BiLSTM-Attention)	0.78
**Ours (CNN-Attention-BiLSTM-Attention)**	**0.82**

**Fig 4:**
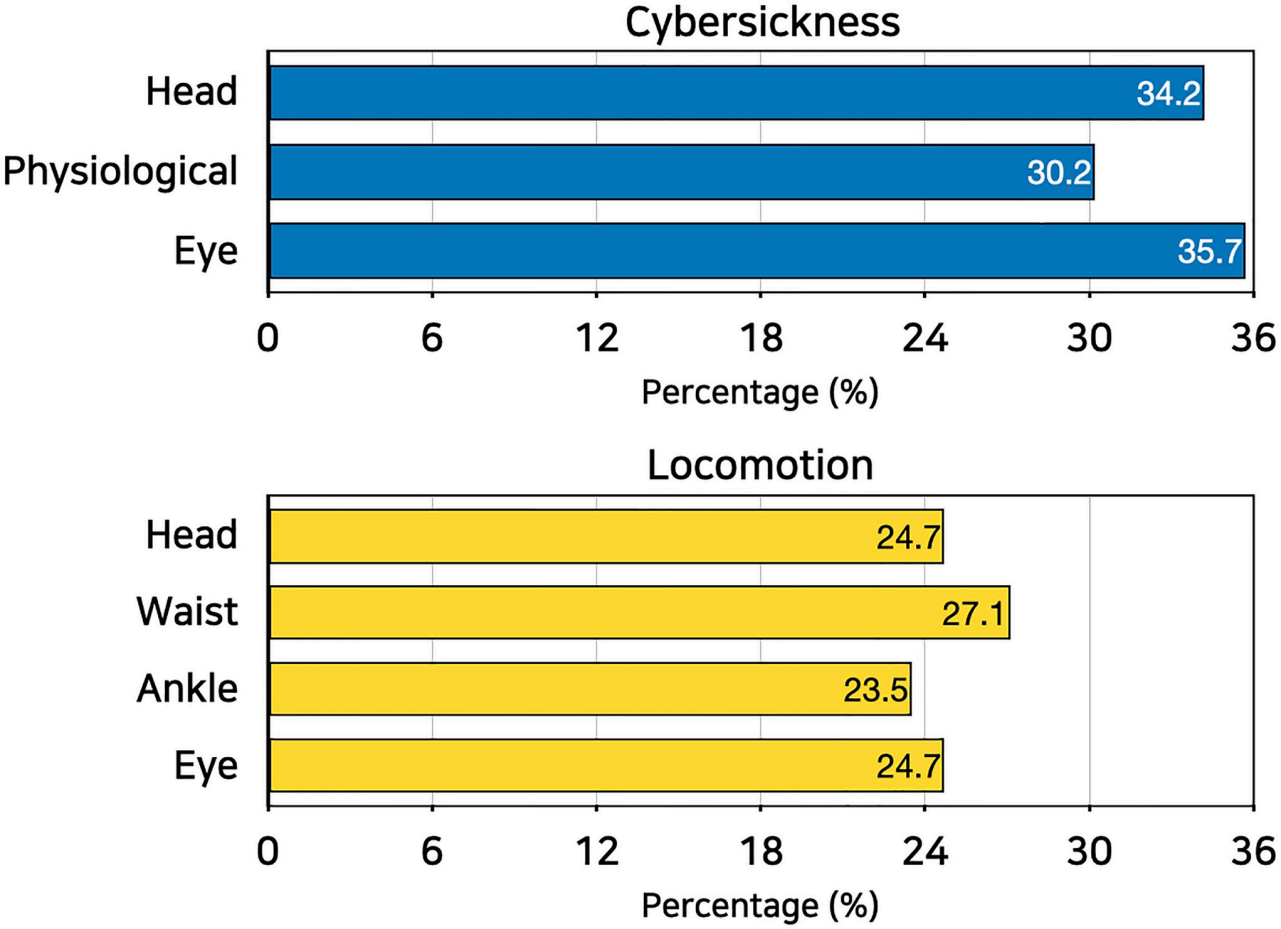
Attention weights of data modalities.

As a result of the ablation study, the role of eye-tracking data is found to be clear. When it is used together with other data modalities, the performance of the model significantly increased (Table 3). Gain indicates the increased ratio of the model performance after the addition of the eye-tracking data (shown in the parenthesis). This result is also well aligned with the result of attention weights placed on each modality (Fig 4-top). Eye-tracking data showed the highest result (35.7%), showing its importance during the model training in the context of cybersickness.

**Table 3:** Ablation study for cybersickness (addition of eye-tracking). Eye-tracking data already shows a strong relationship with cybersickness, and when it is considered with other data modalities, the performance of the model significantly increases. Gain indicates the increased ratio of the model performance after the addition of the eye-tracking data.

Feature groups	F1-score	Gain (%)
Eye	0.68	-
Head (+ Eye)	0.56 (0.75)	33.9
Physiological (+ Eye)	0.36 (0.78)	116.0
[All] Head + Physiological (+ Eye)	0.57 (**0.82**)	43.8

### 6.2 VR locomotion

Table 4 summarizes performance results of the model for the locomotion study. Same as the cybersickness study results, our proposed model yielded the highest performance (0.95 F1-score) compared with other models. The model performances from both studies not only demonstrate the effectiveness of our model architecture (i.e., leveraging attention and temporal characteristics) but also highlight a promising research direction of modeling a user’s states and behaviors in VR.

**Table 4:** The model performance by architecture (locomotion). Our model yielded the highest performance.

Model architecture	F1-score
SVM	0.86
CNN	0.79
BiLSTM	0.85
CNN-BiLSTM	0.90
A-INV (CNN-Attention-BiLSTM)	0.93
A-BiLSTM (CNN-BiLSTM-Attention)	0.94
**Ours (CNN-Attention-BiLSTM-Attention)**	**0.95**

One different point between two studies is a relatively weak role of eye-tracking when only the eye-tracking data was used in modeling. This might be because the second study with locomotion involved a large amount of movements and signals from the participants’ head and body. Thus, it appears that eye-tracking data only did show small influence; yet were used as a strong supplement to improve the learning of locomotion types. This insight is quite well presented in the ablation study. Eye-tracking data shows a comparable attention weight when it is trained together with other sensor modalities (Table 5). The results of the attention weight analysis showed a comparable importance of the eye-tracking data in learning (Fig 4-bottom).

**Table 5:** Ablation study for locomotion (addition of eye-tracking). Using eye-tracking data only shows little influence on locomotion. However, when eye-tracking data are combined with other data modalities, they served as a strong supplement to improve the performance of the model.

Feature groups	F1-score	Gain (%)
Eye	0.15	-
Head (+ Eye)	0.61 (0.80)	31.1
Waist (+ Eye)	0.58 (0.82)	41.3
Ankle (+ Eye)	0.67 (0.74)	10.4
Head + Waist (+ Eye)	0.87 (0.90)	3.4
Head + Ankle (+ Eye)	0.91 (0.89)	2.1
Waist + Ankle (+ Eye)	0.85 (0.91)	7.0
[All] Head + Waist + Ankle (+ Eye)	0.94 (**0.95**)	1.0

## 7 Discussion

One notable aspect of our findings involves the role of multiple sensor data and especially eye-tracking data features obtained from the HMD. When our model was trained with just the HMD data, it performed reasonably similar to its best result (0.75 F1-score from the HMD data vs 0.82 from all data for cybersickness; 0.80 from the HMD data vs 0.95 from all data for locomotion). The HMD is a basic device within the VR/AR sphere, and the fact that the user’s state or behavior can be well understood using only the data from this device is promising but also calls for a more thorough investigation for its extensive application to other VR scenarios. The fact that users can receive support for cybersickness or locomotion issues without wearing additional equipment also emphasizes this data’s high usability.

In this work, we have proven the role of eye-tracking data. Since it has a number of features (e.g., gaze direction, gaze origin, pupil diameter, pupil position, eye openness), as the next step, we believe that it would be important to investigate the role of each feature of the eye-tracking data more extensively. Two approaches can be considered. First, we can measure the attention weight of each feature. Similar to the examination of the attention weight for each data modality, we can extract the attention weight for the features and specifically examine the importance of each feature. Second, we can measure the changes of the eye-tracking data features. Selected, important features can be used in the model to reduce the time for model training and to derive faster model results. This is also expected to enable real-time application of the model to the VR system, which might be one of the requirements for a wider application of the model to many VR scenarios and domains.

Although the role of the attention mechanism was seen to be useful in terms of improving our model performance, we found the influence of the individual subnetwork and the LSTM subnetwork were different between two studies. This indicates that assigning different weights on each subnetwork depending on a VR scenario or goal seems necessary to better learn a degree of a user’s state or behavior. While this insight is interesting, we propose that it may not be generalizable and requires further verification through more experiments. Applying a different approach, such as the Fast Fourier transform [65] (presenting the changes within the energy content of a signal) may improve the characterization of sensor data and produce a better representation thereof through attention. The resulting model would also be more specific to each user upon retraining. As the amount of data used for training for each user increases over time, it may take longer to retrain the model each time. This issue could potentially be mitigated by either applying a weighted model that prefers recency or truncating the past data and applying the most recent data. Lastly, in this work, we trained the model using all participants’ data; though, it is worth considering developing a more personalized model based on only an individual’s data if the amount of the data is sufficient. In summary, there are several potential directions for model development, which is the inspiration for one of our future studies. While the performance of our proposed model has been verified in both scenarios (cybersickness and locomotion), it is also important to examine how good the performance can be in real time. Some VR studies have proved the applicability of the model by comparing the prediction results with the actual values (ground-truth) over time by providing a visualization of the comparison results in real-time [27, 66]. Other studies have reported the performance of the model in a VR environment that is significantly different from the environment in which the data was collected [67]. Although we did not consider the aspect of real-time in this work, we demonstrated high performance of our model in two different VR environments, which also highlights a possible extension of our methodology to learning other types of user states or behaviors that may influence user experience in VR. We believe these are unique contributions compared to other studies, yet will take the real-time aspect of the model into account in our future study.

## 8 Conclusion

This paper examined the role of sensor signals, with a particular focus on eye-tracking data, in the understanding and learning of users’ state or behavior. Although many studies have examined eye-tracking data in the context of user modeling, our study found that the understanding of their role within the virtual environment is somewhat lacking. To fill this gap, we conducted experiments with a total of 50 participants using multimodal sensor data generated during VR experiences in the context of cybersickness (27 participants) and locomotion (23). Our study results highlighted the high feasibility of the methods of using sensor signals to understand users and demonstrated the role that eye-tracking data could play in improving user modeling. The results also showed the effectiveness of our model architecture, which employed the attention mechanism and learned temporal sequences for individual sensor modalities. Important future studies could involve the consideration of more diverse VR situations and users in sensor-based user modeling, the comparison of eye-tracking data to other measurements of users’ state or behavior, and the practical validation of the model through its application to VR systems.
